# YouTube lens to attention deficit hyperactivity disorder: a social media analysis

**DOI:** 10.1186/s13104-018-3962-9

**Published:** 2018-12-04

**Authors:** Pratikshya Thapa, Ashish Thapa, Nabina Khadka, Ruchi Bhattarai, Samir Jha, Amit Khanal, Bibhusan Basnet

**Affiliations:** 1 Kathmandu Medical College, Kathmandu University, Kathmandu, Nepal; 2 Department of Internal Medicine, Presence Saint Joseph Hospital, Chicago, IL USA; 3 Department of Internal Medicine, Bassett Healthcare Network, Cooperstown, NY USA; 4 Department of Internal Medicine, Mount Sinai Hospital, Chicago, IL USA; 5 Department of Internal Medicine, Eastern New Mexico Medical Center, Roswell, NM USA

**Keywords:** YouTube, ADHD, Social media, Attention deficit hyperactivity disorder, Health informatic

## Abstract

**Objective:**

Social media has provided an online environment for patients to discuss regarding their health and seek medical information. The primary aim of our study was to analyze the quality of information shared on YouTube regarding attention deficit hyperactivity disorder (ADHD).

**Results:**

More than half of the videos, 91 (57.23%) had duration of fewer than 5 min. Only 8 (5.03%) videos were rated as highly useful whereas 61 (38.36%) videos were misleading. Interestingly, there was a significant higher (1203.38 ± 395) likes in the misleading group of videos, compared to 162.13 ± 169.63 likes in the very useful group, *P* = 0.012. Only a small fraction of videos had very useful information on ADHD. There is a need for high-quality, evidence-based, educational videos on ADHD for patient education.

**Electronic supplementary material:**

The online version of this article (10.1186/s13104-018-3962-9) contains supplementary material, which is available to authorized users.

## Introduction

Social media presence holds the promise of improving patient education and health literacy [1, 2]. With easy access to the Internet, there has been an exponential use of online resources to seek medical information. With patients and family members becoming increasingly social media-savvy, this participative approach to healthcare to seek medical information is rapidly gaining momentum.

One of the most popular sources of Internet-based medical information is YouTube (http://www.youtube.com). YouTube is a powerful tool for information that is accessed by patients and families with more than 1 billion users [3]. YouTube provides a logical platform for delivery of health information. This website is an open-access site, where registered users can upload video content. Sites such as YouTube represent a powerful tool for the dissemination of information in many formats ranging from personal experiences to professionally made educational material. Unfortunately, the information on this platform is not regulated.

Though there have been various studies in the last decade on quality of information in YouTube videos related to general internal and surgical medicine [4–9]. Limited studies on the quality of these YouTube videos related to mental health including those on anxiety disorder, bullying is published [10, 11]. Attention deficit hyperactivity disorder (ADHD) is a common behavioral disorder in children and adolescent, plus has a significant burden in adult population. Public awareness and education is an integral part of ADHD diagnosis and treatment [12]. Though patients accessing the Internet can be a positive step towards disease treatment and its success, these online resources vary in quality and standard. The primary aim of our study was to conduct a systematic review of the YouTube videos related to ADHD and analyze the quality of information shared on YouTube.

## Main text

### Methods

**Fig. 1:**
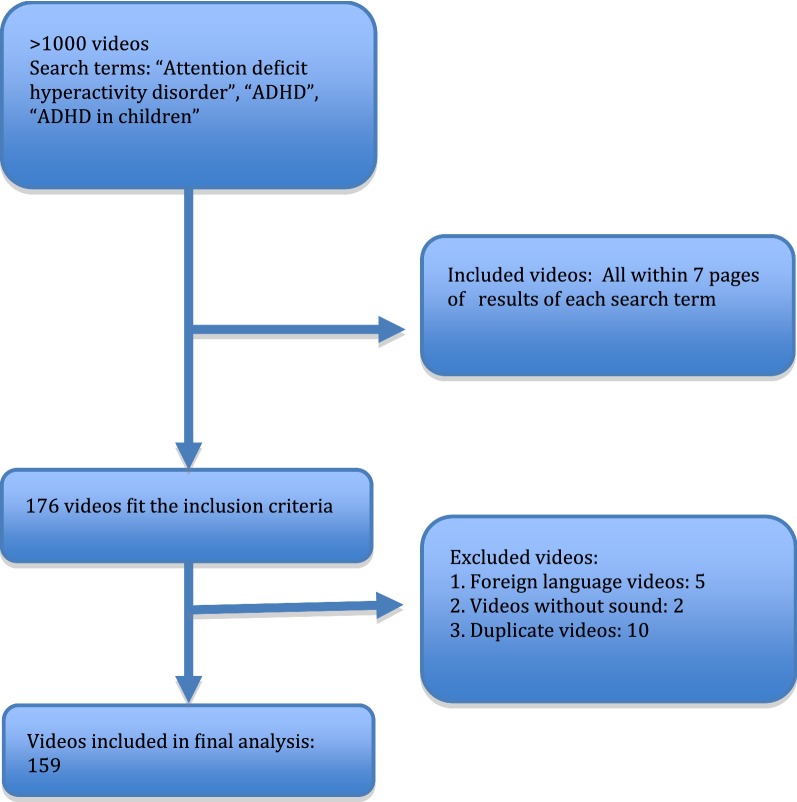
Flow chart showing method of video selection

We conducted a systematic review of the YouTube videos from November 1 to 7, 2017. “Attention deficit hyperactivity disorder”, “ADHD”, “ADHD in children”, was used as keywords to search the video on YouTube. The videos were sorted in the order of number of views. We screened the first 7 pages of results for each search term assuming that users would unlikely scan beyond these pages. This generated 176 videos among which and 159 met the inclusion criteria. Non-English language videos without sound and duplicate videos were excluded from the analysis. The selection process is summarized in Fig. 1. Approval of the Institutional Review Board of the study institution was not required for this study. Two researchers who had completed with MBBS degrees and had sufficient experience in the diagnosis and management of ADHD independently assessed the videos. Disagreements between the researchers regarding the categorization of a particular video were resolved by discussing the issue until a consensus was reached.

Video characteristics from the YouTube site were collected as summary information about the video and viewership. This included the video category, date posted, and length (minutes and seconds). YouTube also collects and provides information to indicate the popularity of video viewing, measured by number of views, number of “like” ratings, number of “dislike” ratings.

The following data were extracted from each video in the final sample:

1. URL, date of upload, duration of video (in seconds).
2. Tube metrics (likes, dislikes, view counts).
3. Source of video upload [Professional societies; Lectures from Medical Institutions; Individual Physician or psychiatrist; personal experience of patients and their families; News reports or other websites and others (non-classified)].
4. Video content: role of parents/teachers mentioned in diagnosis and management; role of cognitive behavioral therapy (CBT) mentioned in diagnosis and management.

The researchers analyzed each YouTube video carefully and looked if the videos had mentioned the role of parents, teachers, medications and cognitive behavioral therapy (CBT). CBT was chosen due to its effective role in treatment for ADHD in adults and children [13]. We devised a scoring system to assess the usefulness of the video. A score of 2.5 each was given for role of parents, teachers, and medications and role of CBT in treatment of ADHD. Scores was summated for each video and Scores between 7.5- 10 was termed “very useful”, 5–7.5 as “useful”, 2.5–5 as “not useful” and < 2.5 as “misleading”.

Data were entered into a Microsoft Excel sheet for analysis (Additional file 1). Statistical analyses were performed with Microsoft excel 2016 and SPSS version 21 (SPSS Inc., Chicago, IL). Basic frequency and descriptive statistics were calculated to describe video characteristics such as media source, topics covered, number of views, and number of days posted to YouTube. Central tendency engagement statistics were computed to determine the median number of days posted to YouTube, “favorites,” “likes,” “dislikes” by media source. Two physician reviewers reviewed all eligible videos, and an inter-observer reliability was tested. A weighted kappa score was calculated to evaluate the inter-observer agreement.

### Results

The inter-observer agreement was 80% with a kappa coefficient of 0.67 (P = 0.001). We found videos on ADHD from personal experience (37, 23.27%), individual physician/psychiatrist (27, 16.98%), institutions/medical society (17, 10.69%), news reports (11, 6.92%). The largest number of videos (50, 31.45%) was uploaded from various online websites unrelated to medical institutions or physician groups and least from commercial sources (5, 3.14%).

Total 159 videos had a mean view of 170,781.65 ± 29,044.41 and mean length of 10.74 ± 1.51 min. Average “like” and “dislike” for 159 videos was 1440.1 ± 348.07 and 73.29 ± 12.17 respectively. These videos were posted for an average duration of 4.5 years with standard error of 2 months. Table 1 contains the demographics of YouTube videos based on sources and engagement metrics. For diagnosis and treatment, the role of parents was mentioned in 63 (39.62%) and role of teachers in 32 (20.13%) of the videos. Only 26 (16.35%) videos, mentioned the role of CBT in the management of the patient. Noteworthy, the discussion of medications in the management of ADHD was mentioned in just 59 (37%) of videos. More than half of the videos, 91 (57.23%) were less than 5 min in length.

**Table 1:** Table with video demographics based on sources and engagement metrics

Source/parameter	Personal experience	Commercial drug companies	Institutions/medical society	Individual physician/psychiatrist	News report	Other websites	Others (non-classified)	Total
Number of videos (%)	37 (23.27%)	5 (3.14%)	17 (10.69%)	27 (16.98%)	11 (6.92%)	50 (31.45%)	12 (7.55%)	159 (100%)
Number of views (± SD)	357,188.4 ± 107,540.04	169,677.2 ± 69,674.7	64,650.76 ± 16,250.36	39,757.11 ± 8182.89	56,432 ± 8646.13	159,828.34 ± 33,815.45	192,104.25 ± 71,048.94	170,781.65 ± 29,044.41
Duration since upload (days) (± SD)	1505.48 ± 162.53	2115 ± 81.78	1673.23 ± 233.47	1805.63 ± 120.34	1432.45 ± 239.44	1816.24 ± 131.28	1210.25 ± 214.17	1663.94 ± 69.58
Opinion								
Number of likes (± SD)	2482.45 ± 874.57	456.2 ± 136.87	201.12 ± 60.9	297.81 ± 110.92	733.81 ± 208.98	1835.86 ± 841.38	1959.91 ± 1075.04	1440.1 ± 348.07
Number of dislikes (± SD)	128.54 ± 37.83	39.8 ± 13.87	17.06 ± 4.9	26.81 ± 14.05	85.81 ± 36.49	64.84 ± 18.77	124.83 ± 54.42	73.29 ± 12.17
Duration of videos (± SD)	6.32 ± 0.93	3.61 ± 1.34	26.25 ± 9.68	10.17 ± 4.04	9.33 ± 2.65	9.83 ± 2.12	11.67 ± 3.99	10.73 ± 1.5
Usefulness Score (± SD)	2.16 ± 0.34	2.5 ± 1.93	2.96 ± 0.94	3.33 ± 0.51	5 ± 0.75	2.45 ± 0.41	3.33 ± 1.03	2.83 ± 0.23
Role of parents mentioned in management (%) (± SD)	12 (32.43%)	2 (40.0%)	7 (41.18%)	13 (48.15%)	8 (72.73%)	15 (30.00%)	6 (50.00%)	63 (39.62%)
Role of teachers mentioned in management (%)	1 (2.7%)	1 (20%)	3 (17.65%)	5 (18.52%)	4 (36.36%)	13 (26.00%)	5 (41.67%)	32 (20.13%)
Role of Medications mentioned in management (%)	14 (37.84%)	1 (20%)	6 (35.29%)	9 (33.33%)	9 (81.82%)	17 (34%)	3 (25%)	59 (37%)
Role of CBT mentioned in management (%)	5 (13.51%)	1 (20%)	4 (2353%)	9 (33.33%)	1 (9.09%)	4 (8%)	2 (16.67%)	26 (16.35%)
Length of videos (min)								
< 5	23	3	11	20	4	27	3	91
5–10	7	2	0	3	4	13	5	34
10–15	4	0	1	2	2	2	3	14
> 15	3	0	5	2	1	8	1	20

Table 2 reflects Video demographics based on usefulness score. Only 8 (5.03%) videos were rated as highly useful, 44 (27.67%) scored useful, 46 (28.93%) videos were not useful and 61 (38.36%) videos were misleading. Among 10 most useful videos, 3 were from institutions and medical society, 2 from various website and one each from physicians, commercial and television source. Videos from news report had the highest usefulness score (5 ± 0.75).

**Table 2:** Table with video demographics based on usefulness score

Relevance/parameters	Very useful	Useful	Not useful	Misleading	P value
Number of videos (%)	8 (5.03%)	44 (27.67%)	46 (28.93%)	61 (38.36%)	
Number of views (± SD)	51,343.5 ± 18,968.64	97,041.61 ± 22,902.16	216,957.37 ± 61,542.78	204,814.16 ± 56,822.95	
Duration since upload in days (± SD)	1581.25 ± 169.63	1668.52 ± 126.47	1577.1 ± 145.28	1736.97 ± 111.27	
Opinion					
Number of likes (± SD)	162.12 ± 57.54	841.7 ± 324.15	2548.65 ± 1026.72	1203.38 ± 395.9	0.012
Number of dislikes (± SD)	18.12 ± 6.71	64.89 ± 19.15	85.15 ± 22.23	77.64 ± 23.19	0.01
Duration of videos (± SD)	24.13 ± 11	11.2 ± 2.56	9.46 ± 3.08	9.6 ± 2.13	0.02
Usefulness score (± SD)	10 ± 0	5.8 ± 0.18	2.5 ± 0	0 ± 0	

*SD* standard deviation

P value denotes significance between very useful and misleading group

Interestingly, there was significant higher likes (1203.38 ± 395.9) in the misleading group of videos compared to 162.12 ± 57.54 likes in the very useful group of videos (P = 0.012). Moreover, the number of dislikes (18.13 ± 6.71) was significantly lower in the very useful group whereas higher dislikes (77.64 ± 23.19) was seen in the misleading group of videos with a P value = 0.01. Videos were significantly long (P value = 0.02) in highly useful group (24.13 ± 11 min) compared to the misleading group (9.60 ± 2.14 min) of videos.

### Discussion

Our survey shows that 61 (38.36%) videos had misleading information while only a small fraction of videos (5.03%) had very useful information on ADHD. We saw that YouTube videos are neither sufficiently comprehensive nor adequately balanced to be recommended as patient education material regarding ADHD. The unregulated nature of YouTube might be partly responsible, in failing to meet a basic standard of information portal. A similar study on gallstone disease reported more than half of the YouTube videos as misleading [4]. An analysis of prostate cancer YouTube videos in 2010 using usefulness score found that 73% of videos had fair or poor content [5]. With patients and family members becoming increasingly social media-savvy, there is a need for high-quality, evidence-based, educational videos on ADHD. If patient and family members are subjected to the low-quality information videos, there is a high risk of these either not finding answers or getting misleading information.

Based on source, the largest number of videos was uploaded from various online websites unrelated to medical institutions or physician groups. A social media analysis on YouTube study of prostate cancer videos found that majority of videos were posted by consumers and medical or government professionals [11]. We observed highest likes in videos posted individual person based on experiences and lowest likes in the group of videos posted by psychiatrist and individual physician. This is likely, as patients tend to trust the information shared by individuals and family dealing with ADHD resulting in more “likes”. The engagement metrics as ‘likes’ and ‘dislikes’ in social media, needs to be evaluated with caution. We are concerned with our findings, which indicated that misleading videos were more popular (more likes and more dislikes) than useful videos. We found similar findings with studies of YouTube videos related to hemodialysis and West Nile virus, which also showed that misleading videos were more popular than useful videos [7–9]. In contrast, we came across several previous studies investigating the quality of YouTube videos on rheumatoid arthritis and varicose veins among others that revealed no difference in the popularity of useful vs. misleading videos [14, 15]. Trusting popularity of videos based on engagement metrics is not completely reliable as it differs widely among medical versus non-medical, educated versus uneducated, and rich versus poor population [16].

We found that Videos from news report had the highest usefulness score. YouTube Videos were of longer duration in highly useful group compared to the misleading group of videos. We can assume that longer videos were found “very useful” as the have enough time to incorporate and explain most aspects of ADHD diagnosis and treatment. We noticed significant higher likes in the misleading group of videos compared to the “very useful” group of videos. Biggs et al. found similar finding in his study and suggested that this is because useful videos tended to be longer than misleading videos [17]. The general population tending to view the misleading videos more than the credible videos is definitely an issue.

### Limitations of the study

1. The quality of information taken into analysis was from one point of time rather than a certain period, thus these results may have changed with time as some videos might have been added or removed.
2. A subjective usefulness score criteria was used to evaluate the videos, as there are as of yet no validated tools for assessing video data.
3. There is the possibility that some videos were not labeled as such and thus were unidentifiable under our search terms, despite all our precautions including conducting the search of YouTube videos independently by two researchers and using four search keywords to compile a common pool.
4. The search in this study was limited to YouTube and there is the possibility that videos on other websites, such as those of medical and other health professional societies and medical journals, were not included.

It’s well accepted that social media can improve patients’ access to health care information and other educational resources [1]. Whether via Twitter, Facebook or YouTube, more healthcare providers and health organizations are embracing social media and patients follow them [18]. Parental and teacher awareness, medication adherence and behavioral therapy are an integral part of ADHD diagnosis and treatment [2, 19]. Social media presence holds the promise of improving patient education and health literacy [18]. In this era of healthcare information technology, the trend of “YouTube search” for an educational purpose has been ever increasing. However, free and unregulated information available on the Internet carries the potential hazard of misinformation. While it is impossible to regulate videos and other sources of information on the Internet, we recommend credible physicians and medical institutions upload videos with accurate information on ADHD. It is the responsibility of professional organizations and healthcare professionals to direct patients to authentic educational resources and advise parents about the inaccurate and potentially misleading content of YouTube and ADHD.

### Additional file

**Additional file 1.** Youtube lens to ADHD videos and its usefulness analysis.

## Appendix

See Tables 1, 2.

## Abbreviations

ADHD: attention deficit hyperactivity disorder
CBT: cognitive behavioral therapy
